# Non-hospital healthcare center’s preparedness assessment toolbar for providing basic emergency care: a sequential exploratory mixed-method study

**DOI:** 10.1186/s12913-023-09053-y

**Published:** 2023-01-23

**Authors:** Homayoun Sadeghi-Bazargani, Mehrdad Amir-Behghadami, Ali Janati, Farzad Rahmani

**Affiliations:** 1 Student Research Committee (SRC), Tabriz University of Medical Sciences, Tabriz, Iran; 2 Road Traffic Injury Research Center, Tabriz University of Medical Sciences, Side to Shahran Tower, Golshahr Square, Eil Goli Street, Tabriz, East Azerbaijan Iran; 3 Iranian Center of Excellence in Health Management, Department of Health Service Management, School of Management and Medical Informatics, Tabriz University of Medical Sciences, Tabriz, Iran; 4 Emergency Medicine Department, Sina Medical Research and Training Hospital, Tabriz University of Medical Sciences, Tabriz, Iran

**Keywords:** Non-hospital healthcare centers, Primary healthcare center, Outpatient clinic, Preparedness, Assessment, Instrument, Toolbar, Emergency care, Life-threatening emergency

## Abstract

**Background:**

Basic emergency management in urban and rural areas is a critical challenge, which can affect the pre-hospital mortality rate. Therefore, Non-hospital Healthcare Center (NHHC) must be prepared to manage such emergency cases that may occur in the geographic area where these centers act. The main aim of the study was to develop and validate an toolbar for NHHCs’ preparedness to provide initial emergency care.

**Methods:**

This study was designed based on a sequential exploratory mixed- method in two phases, in each of which there are three steps. In the phase I, the literature systematic review and qualitative methods (Focus Group Discussions (FGDs) and Semi-Structured Interviews (SSIs)) were applied to identify the domains and items. In the phase II, content validity, feasibility, and reliability of the toolbar were performed. Content validity was assessed using a modified Kappa coefficient based on clarity and relevance criteria. Feasibility of the toolbar was randomly assessed through its implementation in 10 centers in Tabriz. Reliability was randomly assessed in a pilot on 30 centers. Reliability was assessed by measuring internal consistency, test-retest reliability, and inter-rater agreement. The main statistical methods for assessing reliability include Cronbach’s alpha, Intra-class Correlation Coefficient, and Kendal’s Tau-b. All the statistical analyses were performed using Stata 14.

**Results:**

In the phase I, primary version of the toolbar containing 134 items related to assessing the preparedness of NHHCs was generated. In the phase II, item reduction was applied and the final version of the toolbar was developed containing 126 items, respectively. These items were classified in 9 domains which include: “Environmental Infrastructures of Centers”, “Protocols, Guidelines and Policies”, “Medical Supplies and Equipment”, “Emergency Medicines”, “Human Resources”, “Clinical Interventions”, “Maintenance of equipment”, “Medicine Storage Capability”, and “Management Process”. The toolbar had acceptable validity and reliability.

**Conclusions:**

This study provided a standard and valid toolbar that can be used to assess the preparedness of NHHCs to deliver initial emergency care.

**Supplementary Information:**

The online version contains supplementary material available at 10.1186/s12913-023-09053-y.

## Background

Non-hospital health centers (NHHCs) are the most important centers of the health care system and under any circumstances, from delays in the arrival of ambulances to extraordinary situations, as health community centers had to provide basic life support using a minimum of organizational infrastructure [1]. These infrastructures include the physical space, medical equipment, emergency medicines, support facilities, and human forces [2, 3]. Primary health care centers and outpatient clinics, which are the main centers of health care in many rural and urban areas of low-income countries, are a subset of NHHCs [4].

In order to provide integrated care services to individuals, NHHCs in the defined areas are run by health care providers, nurses, and physicians. These providers in Iran are engaged in disease prevention, minor surgery, and the provision of basic emergency care. For this reason, providers must be prepared to deal with life-threatening emergencies (LTEs) that may occur near the geographical area of operation of these centers. They should also cooperate with Emergency Medical Services (EMS) [5]. The first response is a defining event for the patient known as the chain of survival. Primary care must be supported by a strong consultative referral system, which requires collaboration with other organizations [5].

In addition to specialized hospitals, clinics, and polyclinics, NHHCs, including rural and urban health centers, clinics are working to provide primary emergency care to the community, and this is an advantage that can provide the most possible services [6]. These centers facilitate the first access of the injured to the health care system [4]. NHHCs are expected to be prepared to respond to a range of emergency care for patients with chronic and acute cases because life-threatening hazards are constantly occurring suddenly and unexpectedly [7].

At least half of all patients with acute cardiovascular disease have angina pectoris and a quarter are diagnosed with a heart attack [8]. If these people develop cardiac arrest, family physicians, especially in rural areas, are often the first health care professionals to initiate Cardio Pulmonary Resuscitation (CPR) to improve the survival of acute myocardial infarction and reduce mortality. Therefore, providers of primary healthcare centers should be prepared to provide primary emergency services in order to improve the survival of acute myocardial infarction and reduce mortality in such patients [9]. Although out-of-hospital cardiac arrest survival is still poor, it has been observed that those family physicians who acted as cardiac arrest interventions and performed emergency defibrillation referred almost half of the cardiac arrest patients to the hospital. They were successful with a success rate of 12.5%. In addition, 11.6% of serious emergency cases that require resuscitation have been performed by family physicians [10].

The use of emergency care at the level of NHHCs is recommended by several studies as a means to reduce the burden of the hospital emergency department [6, 11–19]. Therefore, providing primary emergency care is not far from expectation [20]. Nevertheless, the question is whether NHHCs have been properly organized to offer emergency care or not. Most of the international studies consistently have reported the unpreparedness of NHHCs for offering emergency care in terms of emergency medicine, equipment, and support facilities [9, 20–25]. Therefore, there is a need to design and develop a toolbar based on specific indicators and criteria for monitoring and evaluation that determine the level of preparedness, shortcomings, and inadequacies in various dimensions of NHHCs and improve the existing situation by modifying them [26].

.A review of the published literature highlights the scarcity of studies on existing research. Although the previous literature has focused mainly on the management of LTEs in hospitals and their preparedness, this issue has been largely ignored in NHHCs [27]. Due to the fact that these centers, as the first level of health care coverage, cover a wider population, so more attention should be paid to the management of LTEs in these centers. It is clear that the existence of valid and reliable toolbar can help identify the capacity of these centers to provide primary emergency care. Some developing as well as developed countries have used non-standard instruments to assess the preparedness of NHHCs to provide primary emergency care, indicating the absence of a comprehensive instrument [6, 10].

The biggest restriction in assessing the preparedness of NHHC in Iran is the lack of a valid and reliable toolbar. Assessing the preparedness of these centers to manage LTEs and provide primary emergency care requires the use of an appropriate and standard instrument [28]. In this regard, either new instruments should be designed by developing psychometric studies or existing instruments should be used. Due to the diverse structure of countries’ health systems, existing instruments may not be fully generalizable to other content, so it is essential that cultural compatibility be considered before using it in new content.

NHHCs can only achieve their goals if they have accurate information about the current situation. The evaluation of these centers has a very important role in providing desirable emergency care to patients [26]. And if the evaluation system of NHHCs is efficient and has tangible and calculable indicators to assess the situation, many costs will be controlled and reduced [1]. The existence of an efficient and effective evaluation toolbar can play an important role in identifying the capabilities of these centers. Based on the best knowledge of researchers, no study has been conducted to design a tool to assess the preparedness of NHHCs to provide primary emergency care. This study aimed to develop and validate a toolbar to NHHCs’ preparedness to provide initial emergency care.

### Research objectives

The objectives of each phase were as follows.

#### The objectives of the qualitative phase

1. Identifying domains and items of the toolbar to assess the preparedness of NHHCs to provide initial emergency care through using a systematic review;
2. Exploring domains and items associated with assessing preparedness from the perspective of experts and target groups; and
3. Defining the final domains and items to be included in the toolbar through triangulating the results of both previous steps by a panel of experts

#### The objectives of the quantitative phase

1. Feasibility of the toolbar through its implementation in 10 NHHCs in Tabriz;
2. Assessing the content validity of the developed toolbar using a modified Kappa coefficient based on clarity and relevance criteria; and
3. Assessing the reliability of the developed toolbar by measuring internal consistency, test-retest reliability, and inter-rater agreement

## Methods and analysis

### Study design

**Fig. 1:**
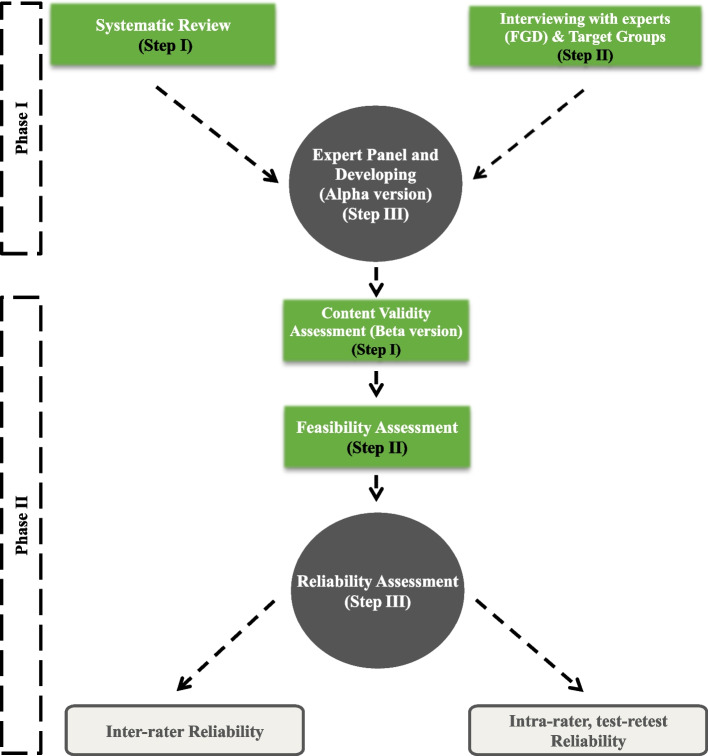
Stages of developing toolbar

The present study was designed based on a sequential exploratory mixed-method in two phases, each of which consisted of three step. Indeed, the sequential exploratory mixed-method was performed in both qualitative and quantitative phases. The first phase was applied to generate items and develop a toolbar, while the second phase was employed to reduce the item and assess psychometric properties (Fig. 1).

The present comprehensive study was conducted to develop a preparedness assessment toolbar for NHHCs to provide basic emergency care. The protocol of this study has already been published in the prestigious journal BMJ Open [29].

#### Phase I: toolbar development

This Phase was carried out in three steps, including a systematic review, FGDs with experts and semi-structured interviews with the target group (emergency care providers and recipients), and a panel of experts. The planned duration for this purpose was 13 months, which was from April 2018 to April 2019.

##### Phase I - step I

In the first step, a comprehensive search strategy was devised to identify the domains and items related to assessing the preparedness of NHHCs, which provide basic emergency care. This systematic review was conducted and reported in accordance with the Preferred Reporting Items for Systematic Review and Meta-analysis (PRISMA). The five databases including PubMed, Scopus, Web of Science, Barakat Knowledge Network Systems (BKNS) and Scientific Information Database (SID) were searched. All of the searches were performed in English and/or Persian languages with no time limit until March, 2018. Also, grey literature and manual search were done. Studies were appraised using the Mixed Methods Appraisal Tool (MMAT). Content and data of the included studies were synthesized by means of directed content analysis methods; then, they were classified according to Donabedian model [30]. For more information on the findings of this step, see the published systematic review and protocol articles [28, 29]. The most comprehensive model used for health care assessment is the Donabedian model. It was presented in 1966 and defined three distinct aspects of quality, which include structure, process, and outcome. This model is more accepted due to its simplicity and flexibility, and most evaluation studies also use this model [31].

##### Phase I - step II

In the second step of toolbar development, in accordance with the health care delivery system in Iran and based on emergency care (basic life support), a qualitative exploratory study was conducted using Semi-Structured Interviews (SSIs) and Focus Group Discussions (FGDs). Those who fail to participate in the FGDs will have a semi-structured interview. Before starting the interviews, the study and its objectives were presented to the participants and their informed consent was secured. SSIs and FGDs were then performed by two skilled researchers using an interview guide developed through literature review and expert consultation. The interviews continued to saturate the data. Overall, 12 SSI were carried out with providers providing care services at NHHC in Tabriz, northwest of Iran. In addition, 2 FGDs were held with 13 specialists in the conference room of the health service management department. The people participating in the FGD sessions were drawn from various specialists, such as Emergency Medicine (EM), Primary Health Care (PHC), the executives of health centers, with over 5 years of work experience, and Emergency Medical Services (EMS) experts. The mean age of the participants was 40 years an average work experience of 13 years. Refer to the table for more details about the participants in the interviews and focus group discussion sessions (Table 1).

**Table 1:** Characteristics of demographic variables of participants in FGD and ISS

Characteristic	Qualitative variables		Number (Percent)
Participations	FGDs with experts	Emergency medicine	4 (16%)
		PHC specialists	2 (8%)
		Executives of health centers	5 (20%)
		EMS experts	2 (8%)
	SSIs with providers	Physicians	6 (24%)
		Nurses	6 (24%)
Gender	Male		20 (80%)
	Female		5 (20%)
Age	30–40		8 (32%)
	41–50		17 (68%)
Work experience (Years)	5–15		15 (60%)
	16–25		10 (40%)
Highest level of educational degree	Bachelor		5 (20%)
	Masters		3 (12%)
	PHD		3 (12%)
	MD, specialist		14 (56%)

Participants were employed using the purposive sampling method because this sampling method ensures that the participants in the interviews have relevant experience and adequate knowledge in the field of research. Snowball sampling was also used to access other physicians with experience in this field. Sampling was continued with maximum variety to achieve greater data transferability of data and saturation [23]. To achieve maximum diversity, informants were selected from different age groups, experiences of dealing with LTEs, and a variety of experiences to provide basic emergency care. All interviews were recorded and transcribed verbatim immediately. As soon as the first interview was performed and transcribed, data analysis was started using the “framework analysis approach”. For more information on the findings of this step, see the published qualitative study and protocol study articles [29, 32].

##### Phase I - step III

In the third step, the domains and items of the relevant toolbar, which were explored through using a systematic review, FGDs, and interviews with the target group, were examined by the research team for overlap and repetition. Then, the table of specifications methodology was used to determine the final domains and items [33]. A panel of emergency medicine experts reviewed items in each domain. In practice, the alignment of a group of items in rows with the existing domains in the columns was examined. Qualitatively, it was authored by a panel of experts based on expert feedback on what other items should be added or removed. Finally, the alpha version of the toolbar was designed.

#### Phase II: item reduction and psychometric evaluation

This phase, as a quantitative phase, included content validity assessment, feasibility, and toolbar reliability. The planned time for this phase was 5 months, which took place from May 2018 to September 2019.

##### Phase II - step I

This step requires the approval of a certain number of specialists, which indicates that the toolbar items and the whole toolbar have valid content. For this purpose, a panel of experts is appointed. The number of experts can not be determined, but at least 5 people are recommended to have sufficient control over the chance agreement. In this regard, an alpha version of the toolbar was sent to 10 selected specialists. The fields of specialty include emergency medicine, PHC specialists, and general practitioners working in clinics and PHC and epidemiologists. In order to calculate the Content Validity Ratio (CVR) of each item, the experts were asked whether each item is essential for the overall performance of the tool. For this purpose, they were asked to rate each item from 1 to 3 with three Likerts “not essential, useful but not essential, essential “. CVR varies between 1 and − 1. A higher score indicates that more panel members agree that an item is essential in a toolbar. Content validation formula in which Ne is the number of experts who selected and rated the “essential” component and N is the total number of people participating in the panel. The numerical value of the CVR was determined by the Lawshe table. For example, in our study of 10 members of the panel of experts, if the CVR is greater than 0.62, the item in the tool would be acceptable with a significant level.

Content validity was assessed using a modified Kappa coefficient (modified CVI). The purpose of the evaluation is to answer the question of whether the content of the toolbar is able to measure the set goals and also to judge the clarity and relevancy of the available items. Finally, experts were asked to provide any questions or other suggestions they had to improve the quality of the questions. A Beta version of the toolbar was prepared. In order to measure the validity of the toolbar, based on the views of experts, CVI and modified KAPA (modified CVI) were evaluated. In addition, in order to consider its content validity index, clarity and relevance, as two criteria, were examined separately on a 4 Likert scale. The modified Kappa coefficient and CVI were calculated for its toolbar and items [34, 35].

To calculate the modified kappa statistic, the probability of chance agreement was calculated for each item by the following formula:

$$\textrm{PC}=\left[\textrm{N}!/\textrm{A}!\left(\textrm{N}-\textrm{A}\right)!\right]\ast .5\textrm{N}$$

In this formula, N stands for the number of experts and, A stands for the number of agreeing specialists.

After calculating the content validity index for each item for all tool items, kappa was computed by including the numerical values of probability of chance agreement (PC) and content validity index of each item (I-CVI) in the following formula:

$$\textrm{K}=\left(\textrm{I}-\textrm{CVI}-\textrm{PC}\right)/\left(1-\textrm{PC}\right)$$

Kappa evaluation criteria, using the guidelines described in Cicchetti, Sparrow and Fleiss, are:

Fleiss (1981) is: Excellent = k > .74; Good = k of .60–.74; and Fair = k of .40 to .59 [36].

##### Phase II - step II

In the second step, the feasibility of NHHCs preparedness assessment toolbar regarding the simplicity of answering toolbar questions and other possible problems in 10 NHHCs in Tabriz was randomly evaluated. Then, the necessary changes and corrections were made.

##### Phase II - step III

In the last step of phase II, two Intra-rater and Inter-rater reliability assessment methods were used to evaluate the reliability of the toolbar.

- Intra-rater، test-retest:

It evaluates the stability of measurements over time. To assess the internal reliability of the developed toolbar, the preparedness of NHHCs was assessed by an assessment team. The evaluators also re-evaluated the same NHHCs to achieve internal reliability. Depending on the issue, the test-retest period was considered 2 weeks after the first assessment.

Also, to calculate internal reliability, the assessment team re-evaluated the same NHHCs after a period of time from their first assessments.

- Inter-rater agreement

To evaluate the inter-rater reliability, NHHCs were divided into two parts and evaluated separately by two evaluators. At this step of the research, the NHHCs were evaluated by a team of physicians and experts.

### Sample size

For the pilot test, the minimum sample consisted of 30 NHHCs, and according to the diversity of these centers and in terms of related conditions, stratified random sampling was used. The sample framework for selecting categories was based on the list obtained from the vice chancellery for health.

### Analysis

All statistical analysis were performed using Stata 14 statistical software package. Toolbar reliability was assessed through internal consistency, as well as the test-retest and inter-rater reliability methods. Cronbach’s alpha was used to measure internal consistency for the whole toolbar and each sub-domain. For test–retest reliability of the toolbar, the Intra-class Correlation Coefficient (ICC) in the time interval was used to calculate the whole toolbar and each sub-domain. The internal consistency was measured by the coefficient Cronbach’s alpha, which varies from 0 to 1, and values equal to or > 0.70 for a scale show a satisfactory internal consistency [37]. Internal consistency reliability and ICC were classified and interpreted into 6 categories [38]. (Table 2). In addition, Kendal’s Tau-b correlation coefficients were reported for the reliability of the test-retest.

**Table 2:** ICC classification criteria for its interpretation

Confidence value for ICC	Interpretation
0<	Less than chance agreement
0.01–0.20	Slight agreement
0.21–0.40	Fair agreement
0.41–0.60	Moderate agreement
0.61–0.80	Substantial agreement
0.81–0.99	Almost perfect agreement

## Results

### Phase I - step I

Out of 3014 studies, 15 studies were included for data synthesis. Our results presented 15 main domains and 25 subdomains. Then, they were classified based on Donabedian’s triple model and a conceptual framework was developed. Out of the 15 included studies, 1 study considered 10 synthesized domains, and 14 other studies considered 4–8 domains of all the 15 synthesized domains. In these studies, the most prevalent synthesized domains were “medical supplies and equipment” and “human resources”, which were considered in 15 studies، “educating and training”, were considered in 14 studies. And, “support facilities” and “emergency medicines” were considered in 13 studies; while, “infrastructures” were considered in 8 studies. *Refer to the published article for more details on the findings of the systematic review section* [28]*.*

### Phase I - step II

According to Donabedian’s triple model, the findings of the interviews were divided into 3 themes and 11 sub-themes. The domains affecting NHHC’s preparedness to provide basic emergency care included input, process, and outcome, in which there were 5, 4, and 2 sub-themes respectively. Input was in 5 sub-themes, including medical equipment and supplies, environmental infrastructures of the centers, emergency medicines, human resources, and protocols, guidelines and policies; process was in 4 sub-themes, including providing clinical services, medicine storage capacity, maintenance of equipment, and management process; finally, outcome was in 2 sub-themes, including patients’ satisfaction with the quality of care and improved survival of LTE patients. All data were extracted from the participant’s points of view. *Refer to the published article for more details on the findings of the qualitative study section* [32]*.*

### Phase I - step III

Resource allocation in low-income and developing countries is often weak, and the lack of the most basic resources is a challenge that is emphasized by the findings of the systematic review as well as other studies conducted in this field [5, 28, 32]. Therefore, it is recommended to pay more attention to the domains of input and process when assessing the preparedness of NHHCs. The research team identified only the input and process areas as appropriate for tool design. However, they do not deny the need for the effects of outcomes in measuring the preparednss of centers. In addition, during two 2-hour sessions, the research team reviewed the domains and items of the toolbar for overlap and replication, and then prepared some initial demographic information for the toolbar. After evaluating the panel of experts using the methodology table of specifications, alpha versions of the toolbar were finally designed. The NHHCs preparedness assessment toolbar was designed in 2 main domains, 10 sub-domains of 134 items. During the discussion on how to rate the items, the research team in the second session prepared an initial guide for the toolbar to help and guide the evaluators in the evaluations.

### Phase II - step I

After selecting 10 experts, a specialized panel was created. To make quantitative and qualitative decisions about the tool, panel members were asked to judge the content validity ratio, the content validity index, and the comprehensiveness of the tool. The letter of request, which included a brief explanation of the subject and objectives of the study, the scoring method, and the necessary instructions for responding, was mostly sent in person and by e-mail. Theoretical definitions of the basic study of input, its dimensions, and items of each area are also mentioned in that letter.

In the NHHCs preparedness assessment toolbar for providing primary emergency care, 8 items out of 134 items were removed. Deleted items in the toolbar had a CVR of less than 0.62 (according to the number of experts in our study, which was 10, the numerical values of the table was equal to 0.62) (Table 3).

**Table 3:** The CVR, CVI and modified Kappa for each item

The CVR, CVI and modified Kappa for each item											
**Sub-domains and Items**	**Essential**			**Relevance**				**Clarity**			
	**Ne**^a^	**CVR**^b^	**Interpretation**	**Number giving rating of 3 or 4 to relevancy of item**	**CVI**^c^	**p**_c_^d^	**Modified Kappa (Modified CVI)**	**Number giving rating of 3 or 4 to clarity of item**	**CVI**	**p**_c_	**Modified Kappa (Modified CVI)**
**S-d 1: Environmental Infrastructures of the Centers**											
S-d 1–1	9	0.80	Remained	9	1	0.001	1	9	0.9	0.009	0.89
S-d 1–2	10	1	Remained	8	0.8	0.043	0.79	9	0.9	0.009	0.89
S-d 1–3	10	1	Remained	9	0.9	0.009	0.89	9	0.9	0.009	0.89
S-d 1–4	10	1	Remained	8	0.8	0.043	0.79	9	0.9	0.009	0.89
**S-d 2: Protocols, Guidelines and Policies**											
S-d 2–1	9	0.80	Remained	9	0.9	0.009	0.89	9	0.9	0.009	0.89
S-d 2–2	10	1	Remained	9	0.9	0.009	0.89	9	0.9	0.009	0.89
S-d 2–3	10	1	Remained	9	0.9	0.009	0.89	8	0.88	0.017	0.88
S-d 2–4	10	1	Remained	9	0.9	0.009	0.89	9	0.9	0.009	0.89
S-d 2–5	9	0.8	Remained	9	1	0.001	1	9	1	0.001	1
S-d 2–6	9	0.8	Remained	8	0.88	0.017	0.88	8	0.88	0.017	0.88
S-d 2–7	10	1	Remained	8	0.88	0.017	0.88	8	0.88	0.017	0.88
**S-d 3: Medical Supplies and Equipment**											
S-d 3–1	10	0.80	Remained	6	0.75	0.109	0.71	6	0.85	0.054	0.84
S-d 3–2	10	1	Remained	7	0.87	0.031	0.87	8	0.8	0.043	0.79
S-d 3–3	10	1	Remained	8	0.8	0.043	0.79	7	0.87	0.031	0.87
S-d 3–4	10	1	Remained	8	0.88	0.017	0.88	8	0.88	0.017	0.88
S-d 3–5	10	1	Remained	9	0.9	0.009	0.89	9	0.9	0.009	0.89
S-d 3–6	9	0.8	Remained	9	0.9	0.009	0.89	9	0.9	0.009	0.89
S-d 3–7	10	1	Remained	9	1	0.001	1	9	0.9	0.009	0.89
S-d 3–8	9	0.8	Remained	8	0.88	0.017	0.88	9	0.9	0.009	0.89
S-d 3–9	10	1	Remained	8	0.88	0.017	0.88	9	0.9	0.009	0.89
S-d 3–10	10	1	Remained	8	0.88	0.017	0.88	9	1	0.001	1
S-d 3–11	10	1	Remained	9	1	0.001	1	8	0.88	0.017	0.88
S-d 3–12	10	1	Remained	8	0.8	0.043	0.79	8	0.88	0.017	0.88
S-d 3–13	10	1	Remained	8	0.8	0.043	0.79	9	0.9	0.009	0.89
S-d 3–14	10	1	Remained	8	0.88	0.017	0.88	9	1	0.001	1
S-d 3–15	10	1	Remained	9	1	0.001	1	8	0.88	0.017	0.88
S-d 3–16	10	1	Remained	8	0.8	0.043	0.79	8	0.88	0.017	0.88
S-d 3–17	10	1	Remained	9	0.9	0.009	0.89	9	0.9	0.009	0.88
S-d 3–18	9	0.8	Remained	7	1	0.007	1	8	0.88	0.017	0.87
S-d 3–19	10	1	Remained	9	1	0.001	1	8	0.8	0.043	0.79
S-d 3–20	10	1	Remained	8	0.88	0.017	0.88	9	1	0.001	1
S-d 3–21	10	1	Remained	8	0.88	0.017	0.88	8	0.88	0.017	0.88
S-d 3–22	10	1	Remained	7	1	0.007	1	9	1	0.001	1
S-d 3–23	10	1	Remained	9	1	0.001	1	8	0.88	0.017	0.88
S-d 3–24	10	1	Remained	9	0.9	0.009	0.89	9	0.9	0.009	0.89
S-d 3–25	10	1	Remained	9	0.9	0.009	0.89	9	0.9	0.009	0.89
S-d 3–26	10	1	Remained	9	1	0.001	1	9	1	0.001	1
S-d 3–27	9	0.8	Remained	9	0.9	0.009	0.89	9	1	0.001	1
S-d 3–28	9	0.8	Remained	9	0.9	0.009	0.89	8	0.8	0.043	0.79
S-d 3–29	9	0.8	Remained	9	0.9	0.009	0.89	8	0.88	0.017	0.88
S-d 3–30	10	1	Remained	9	1	0.001	1	9	1	0.001	1
S-d 3–31	10	1	Remained	9	0.9	0.009	0.89	9	0.9	0.009	0.89
S-d 3–32	10	1	Remained	9	1	0.001	1	7	1	0.007	1
S-d 3–33	10	1	Remained	9	0.9	0.009	0.89	9	1	0.001	1
S-d 3–34	10	1	Remained	8	0.88	0.017	0.88	9	1	0.001	1
S-d 3–35	10	1	Remained	8	0.88	0.017	0.88	9	0.9	0.009	0.89
S-d 3–36	9	0.8	Remained	9	0.9	0.009	0.89	8	0.8	0.043	0.79
S-d 3–37	9	0.8	Remained	8	0.88	0.017	0.87	8	0.8	0.043	0.79
S-d 3–38	10	1	Remained	9	1	0.001	1	9	1	0.001	1
S-d 3–39	10	1	Remained	8	0.88	0.017	0.88	9	0.9	0.009	0.89
S-d 3–40	10	1	Remained	8	0.88	0.017	0.88	8	0.88	0.017	0.88
S-d 3–41	10	1	Remained	9	0.9	0.009	0.89	8	0.88	0.017	0.88
S-d 3–42	10	1	Remained	9	1	0.001	1	9	0.9	0.009	0.89
S-d 3–43	9	0.8	Remained	9	0.9	0.009	0.89	8	0.88	0.017	0.88
S-d 3–44	10	1	Remained	8	0.88	0.017	0.88	6	0.75	0.109	0.71
S-d 3–45	10	1	Remained	6	0.75	0.109	0.71	9	1	0.001	1
S-d 3–46	10	1	Remained	7	0.87	0.031	0.87	6	0.75	0.109	0.71
S-d 3–47	10	1	Remained	7	0.87	0.031	0.87	7	0.87	0.031	0.87
S-d 3–48	10	1	Remained	8	0.8	0.043	0.79	6	0.75	0.109	0.71
S-d 3–49	10	1	Remained	5	0.71	0.164	0.65	6	0.66	0.164	0.6
S-d 3–50	10	1	Remained	6	0.66	0.164	0.6	5	0.71	0.164	0.65
S-d 3–51	9	0.8	Remained	6	0.66	0.164	0.6	6	0.66	0.164	0.6
S-d 3–52	9	0.8	Remained	9	0.9	0.009	0.89	8	0.88	0.017	0.88
S-d 3–53	10	1	Remained	8	0.88	0.017	0.88	6	0.75	0.109	0.71
S-d 3–54	10	1	Remained	5	0.71	0.164	0.65	8	0.88	0.017	0.88
S-d 3–55	10	1	Remained	8	0.88	0.017	0.88	7	1	0.007	1
S-d 3–56	10	1	Remained	8	0.88	0.017	0.88	9	1	0.001	1
S-d 3–57	10	1	Remained	6	0.75	0.109	0.71	7	0.87	0.031	0.87
S-d 3–58	10	1	Remained	7	0.87	0.031	0.87	7	0.87	0.031	0.87
**S-d 4: Human Resources**											
S-d 4–1	10	1	Remained	8	0.88	0.017	0.87	9	0.9	0.009	0.89
S-d 4–2	10	1	Remained	9	1	0.001	1	9	1	0.001	1
S-d 4–3	10	1	Remained	8	0.88	0.017	0.88	9	1	0.001	1
**S-d 5: Emergency Medicines**											
S-d 5–1	10	1	Remained	9	0.9	0.009	0.89	9	1	0.001	1
S-d 5–2	10	1	Remained	9	1	0.001	1	8	0.88	0.017	0.88
S-d 5–3	10	1	Remained	9	0.9	0.009	0.89	9	1	0.001	1
S-d 5–4	10	1	Remained	9	1	0.001	1	8	0.88	0.017	0.88
S-d 5–5	4	−0.2	Eliminated	–	–	–	–	–	–	–	–
S-d 5–6	9	0.8	Remained	9	0.9	0.009	0.89	9	0.9	0.009	0.89
S-d 5–7	10	1	Remained	8	0.88	0.017	0.87	9	1	0.001	1
S-d 5–8	10	1	Remained	8	0.8	0.043	0.79	8	0.88	0.017	0.88
S-d 5–9	9	0.8	Remained	9	1	0.001	1	8	0.88	0.017	0.88
S-d 5–10	10	1	Remained	8	0.88	0.017	0.88	9	0.9	0.009	0.89
S-d 5–11	10	1	Remained	9	0.9	0.009	0.89	9	0.9	0.009	0.89
S-d 5–12	10	1	Remained	9	0.9	0.009	0.89	9	0.9	0.009	0.89
S-d 5–13	10	1	Remained	8	0.88	0.017	0.88	9	0.9	0.009	0.89
S-d 5–14	10	1	Remained	9	0.9	0.009	0.89	9	1	0.001	1
S-d 5–15	10	1	Remained	9	1	0.001	1	8	0.88	0.017	0.88
S-d 5–16	9	0.8	Remained	9	0.9	0.009	0.89	9	0.9	0.009	0.89
S-d 5–17	9	0.8	Remained	8	0.88	0.017	0.88	8	0.88	0.017	0.88
S-d 5–18	9	0.8	Remained	9	0.9	0.009	0.89	9	1	0.001	1
S-d 5–19	9	0.8	Remained	9	1	0.001	1	8	0.8	0.043	0.79
S-d 5–20	9	0.8	Remained	8	0.88	0.017	0.88	8	0.8	0.043	0.79
S-d 5–21	9	0.8	Remained	9	0.9	0.009	0.89	9	0.9	0.009	0.89
S-d 5–22	9	0.8	Remained	9	0.9	0.009	0.89	9	0.9	0.009	0.89
S-d 5–23	9	0.8	Remained	8	0.88	0.017	0.88	9	0.9	0.009	0.89
S-d 5–24	9	0.8	Remained	9	0.9	0.009	0.89	9	0.9	0.009	0.89
S-d 5–25	9	0.8	Remained	9	1	0.001	1	9	1	0.001	1
S-d 5–26	9	0.8	Remained	8	0.88	0.017	0.88	9	0.9	0.009	0.89
S-d 5–27	10	1	Remained	9	0.9	0.009	0.89	9	1	0.001	1
S-d 5–28	9	0.8	Remained	8	0.88	0.017	0.88	9	0.9	0.009	0.89
S-d 5–29	10	1	Remained	8	0.88	0.017	0.88	9	1	0.001	1
S-d 5–30	10	1	Remained	7	1	0.007	1	8	0.88	0.017	0.88
S-d 5–31	10	1	Remained	9	1	0.001	1	8	0.88	0.017	0.88
**S-d 6: Providing Clinical Services For Resuscitation**											
S-d 6–1	10	1	Remained	8	0.88	0.017	0.88	9	0.9	0.009	0.89
S-d 6–2	10	1	Remained	9	0.9	0.009	0.89	8	0.8	0.043	0.79
S-d 6–3	10	1	Remained	8	0.88	0.017	0.87	8	0.8	0.043	0.79
S-d 6–4	10	1	Remained	9	1	0.001	1	9	1	0.001	1
S-d 6–5	10	1	Remained	9	0.9	0.009	0.89	8	0.8	0.043	0.79
S-d 6–6	10	1	Remained	8	0.88	0.017	0.87	8	0.8	0.043	0.79
S-d 6–7	10	1	Remained	8	0.8	0.043	0.79	9	0.9	0.009	0.89
S-d 6–8	9	0.8	Remained	8	0.8	0.043	0.79	8	0.88	0.017	0.87
S-d 6–9	10	1	Remained	9	1	0.001	1	9	1	0.001	1
S-d 6–10	10	1	Remained	8	0.8	0.043	0.79	9	0.9	0.009	0.89
S-d 6–11	9	0.8	Remained	8	0.8	0.043	0.79	9	0.9	0.009	0.89
S-d 6–12	10	1	Remained	8	0.8	0.043	0.79	9	0.9	0.009	0.89
**S-d 7: Clinical Interventions For Specific Injuries**											
S-d 7–1	4	−0.2	Eliminated								
S-d 7–2	7	0.4	Eliminated								
S-d 7–3	7	0.4	Eliminated								
S-d 7–4	2	−0.6	Eliminated								
S-d 7–5	7	0.4	Eliminated								
S-d 7–6	4	−0.2	Eliminated								
**S-d 8: Medicine Storage Capability**											
S-d 8–1	9	0.8	Remained	8	0.8	0.043	0.79	9	0.9	0.009	0.89
S-d 8–2	10	1	Remained	8	0.8	0.043	0.79	8	0.88	0.017	0.87
S-d 8–3	9	0.8	Remained	8	0.8	0.043	0.79	8	0.8	0.043	0.79
S-d 8–4	9	0.8	Remained	8	0.88	0.017	0.87	8	0.8	0.043	0.79
S-d 8–5	10	1	Remained	9	0.9	0.009	0.89	9	0.9	0.009	0.89
S-d 8–6	9	0.8	Remained	8	0.8	0.043	0.79	8	0.8	0.043	0.79
**S-d 9: Maintenance of Equipment**											
S-d 9–1	9	0.8	Remained	9	0.9	0.009	0.89	9	0.9	0.009	0.89
S-d 9–2	10	1	Remained	8	0.88	0.017	0.87	8	0.88	0.017	0.87
S-d 9–3	9	0.8	Remained	8	0.8	0.043	0.79	9	1	0.001	1
**S-d 10: Management Process**											
S-d 10–1	9	0.8	Remained	8	0.8	0.043	0.79	9	1	0.001	1
S-d 10–2	10	1	Remained	8	0.8	0.043	0.79	8	0.88	0.017	0.88
S-d 10–3	9	0.8	Remained	8	0.8	0.043	0.79	8	0.88	0.017	0.88
S-d 10–4	10	1	Remained	9	0.9	0.009	0.89	9	0.9	0.009	0.89
S-d 10–5	9	0.8	Remained	9	1	0.001	1	9	1	0.001	1
S-d 10–6	10	1	Remained	9	0.9	0.009	0.89	8	0.8	0.043	0.79

*NOTE*:^a^Number of experts evaluated the item essential

^b^CVR or Content Validity Ratio = (Ne-N/2)/(N/2) with 10 person at the expert panel (*N* = 10), the items with the CVR bigger than 0.62 remained at the instrument and the rest eliminated

^c^CVI or Content Validity Index

^d^pc (probability of a chance occurrence) was computed using the formula: pc = [N! /A! (N -A)!]

Deleted items include:

- ◾ The deleted item in the sub-domains of emergency medicine sub-domains was “morphine”.
- ◾ The omitted item in the sub-domains of clinical services for resuscitation was “airway risk assessment”.
- ◾ Deleted items of the seventh sub-domain that were clinical interventions for specific injuries. Items in this sub-domain also include the following:
-
  - ◾ Preliminary actions when head injuries
  - ◾ Preliminary actions when trauma to the neck
  - ◾ Preliminary actions when traumatic injuries to the chest
  - ◾ Preliminary actions when traumatic injuries to the abdomen
  - ◾ Preliminary measures for fractures, spinal cord injuries and multiple traumas
  - ◾ Preliminary measures for wounds and burns

The content validity of the tool was evaluated based on both qualitative methods (panel members’ opinions) and quantitative (level of agreement of experts panel members that the content validity index and modified kappa were 0.88 and 0.88 for relevance and 0.89 and 0.89 for clarity, respectively). 13 items were modified without deleting any items based on the recommendations of the panel of experts and members of the research team. Also, the opinions of some members of the panel of experts regarding the guidance of evaluation toolbar were changed and modified after consultation and discussion with the research team. Finally, beta versions of the instument were prepared with 9 subdomains and 126 items (Table 1).

Toolbar items that were modified based on the comments and suggestions of expert members include:

- ◾ Modified items in the domain of “physical space of centers” include:
  - The item “Existence of a protective ramp” was changed to “Existence of a protective ramp with a slope of 7% so that a stretcher or wheelchair can be placed in it and in front of the center entrance door”.
  - The item “Existence of a separate location for managing threatening emergencies” was changed to “Existence of a dedicated location on the ground floor near the center entrance door for emergency care”.
  - The item “Existence of a waiting room” was changed to “Existence of a waiting room that is managed continuously and regularly under the supervision of a clinical staff member”.
- ◾ Modified item in the domain of guidelines, protocols and policies:
  - The item “Existence of policies on equipment calibration” was changed to “Existence of written policy and procedure indicating how medical equipment is used and maintained”.
- ◾ Modified items in the domain of medical equipment and supplies including:
  - The “suction” item was changed to “portable suction with acceptable suction (plus central suction), suction interface and yankauer suction tip “.
  - The item “Infant and pediatric laryngoscope” was changed to “Infant and pediatric laryngoscope with blades in different sizes of curved and smooth (with 2 healthy spare batteries and one laryngoscope spare lamp)”.
  - The “adult laryngoscope” item was changed to “adult laryngoscope with different curved and smooth sizes (with 2 healthy spare batteries and one laryngoscope spare lamp)”.
  - The “Oxygen Capsule” item was changed to “Oxygen Capsule with capsule carrier wheel and protective chain and oxygen interface tube”.
  - “The “Spirometer“ item was changed to “Peak Flowmeter “because the use of courier flowmeter has recently become common in centers”.
  - The “Emergency Trolley” item was changed to “Emergency Trolley is available to clinical staff so that resuscitators can use its contents in less than one minute”.
  - The “Oxygen Mask” item was changed to “Disposable Facial Oxygen Mask with Interface Tube in the sizes of children and adults, at least one number each”.
- ◾ Modified item in the field of resuscitation management processes:
  - The item “Measurement of severity, triage and decision making of emergency measures in the form of ALS” was changed to “Registering the severity of injury (At least as (GAP) (GCS, Age & Pluse p))”.
- ◾ Modified item in the domain of emergency medicine stock monitoring:
  - The item “Existence of routine schedule for requesting emergency medicines” was changed to “Existence of routine schedule for requesting pharmaceutical items from relevant organizations or supply by the centers themselves”.

According to the panel of experts, the rating of items related to the areas of “physical space of centers” and “guidelines, protocols and policies” from Likert is three points “Not available, sometimes, always” were changed to 2 points “does not exist and exists”. These domains are from items 1 to 11. Also, items related to the domain of medical equipment in the form of 4 points, (“0: does not exist”, “1: exists but cannot be used (broken)”, “2: has no problem”, “3: exists, is not ready for fast consumption”, “4: exists, ready for fast consumption “) which was changed to three parts 2 points, 3 points and 4 points that can be seen in the tool guide. Items 12 to 68 are related to this domain.

### Phase II - step II

Some items of the toolbar were not feasible in terms of accessibility and the possibility of preparation during evaluations in NHHCs of Iran. Since the findings of the systematic review and qualitative interviews with experts and providers show the minimum items required when assessing the preparedness of NHHCs, so the items included in the toolbar should be observed and evaluated based on them. For example, some items in the field of “medical equipment and supplies” such as electrocardiography, radiology, ultrasound, oxygen capsule with capsule carriers and protective chains and oxygen interface tubes, portable suction with acceptable suction (in addition to central suction), yankauer suction tip and suction interface, laryngoscope for infants, children and adults with blades in different curved and smooth sizes (with 2 healthy spare batteries and one laryngoscope spare lamp), disposable oral airway and nasal airway Once used, these items were not available in most of these 4 centers, so evaluations were not possible, but this does not mean that these items should be removed because they are not available in any of the centers. Domains and items included in this toolbar are used to assess the preparedness of NHHCs to provide primary emergency care. The vice chancellery for the health of East Azerbaijan Province will be able to design and implement interventions to improve the preparedness of these centers at the local and national levels after the evaluations made using this toolbar.

### Phase II - step III

The internal consistency and test-retest reliability were shown in Table 2. The final toolbar is attached in Supplementary file 1. The Cronbach’s alpha, ICC and Kendal’s Tau-b correlation coefficients for the toolbar were 0.95, 0.76 and 0.78 respectively (Table 4) .

**Table 4:** Cronbach’s alpha and reliability of test-retest

Sub-domains	Number of items	Cronbach’s alpha	ICC^a^ (95%confidence interval)	Test-retest reliability	Kendall’s tau-b
**Environmental Infrastructures of Centers**	4	0.66	0.68	Substantial agreement	0.78
**Protocols, Guidelines and Policies**	7	0.70	0.77	Substantial agreement	0.81
**Medical Supplies and Equipment**	57	0.98	0.98	Almost perfect agreement	0.78
**Human Resources**	3	0.81	0.95	Almost perfect agreement	0.77
**Emergency Medicines**	30	0.84	0.93	Almost perfect agreement	0.75
**Clinical Interventions**	12	0.89	0.81	Almost perfect agreement	0.76
**Medicine Storage Capability**	6	0.88	0.87	Almost perfect agreement	0.83
**Maintenance of equipment**	3	0.55	0.83	Almost perfect agreement	0.79
**Management Process**	6	0.79	0.86)	Almost perfect agreement	0.78

*NOTE*: ^a^ICC or Intra-class Correlation Coefficient

## Discussion

The purpose of this study was to design and evaluate the psychometric properties of the NHHC preparedness measuring toolbar for providing primary emergency care. An important part of the present study was the review of previous studies that assess the preparedness of NHHCs. A systematic literature review showed us that very few studies have been conducted in this regard and that there are no standard and comprehensive tools for evaluations [28]. However, each study showed us which domains and factors can play a key role in the preparedness of centers and affect their ability to manage LTEs. The results of this study showed that the designed toolbar is valid and reliable. In addition, Cronbach’s alpha coefficient and their internal correlation coefficient were acceptable and showed that the toolbar has good reliability. The tool consisted of 126 items in 9 sub-domains and two input and process dimensions. These sub-domains include “Environmental Infrastructures of Centers”, “Protocols, Guidelines and Policies”, “Medical Supplies and Equipment”, “Emergency Medicines”, “Human Resources”, “Clinical Interventions”, “Maintenance of Equipment”, “Medicine Storage Capability”, and “Management Process”.

Input as a relatively sustainable concept includes the characteristics created by resources such as human resources, and infrastructure related to physical space, medical equipment and supplies [39], so it can affect the performance and readiness of NHHCs [40].

The first sub-domain in the input dimension includes 4 items related to the environmental infrastructure of centers, which must be considered to meet the emergency needs of the patients covered, an environment that can provide life-saving measures in the fastest possible time and with maximum efficiency. Therefore, paying attention to the location of the centers and designing the internal and external environment of the centers in accordance with the standards is very important for the level of preparedness of NHHC s. In the evaluation of infrastructure, it is necessary to pay attention to the physical space to address the emergency needs of patients, a space that can provide the necessary facilities in the shortest and fastest possible time with maximum efficiency and comply with the standard of NHHCs [19]. Therefore, it is very important to pay attention to components such as the emergency room on the same floor and near the entrance door in health centers to assess the preparedness of NHHCs [4, 6, 19, 41–44].

The second sub-domain in the input dimension, with 7 items related to protocols, guidelines and policies. Protocols and guidelines such as the maintenance of medical equipment lead to quality assurance of care. Similarly, Razzak et al. referred to the treatment protocol in the early management of acute coronary syndrome and the treatment of patients with multiple traumas [45]. Also, Mohey et al. reported that written guidelines for primary emergency care [43], pediatric emergency triage guidelines, assessment, and treatment [43] should be available at non-hospital facilities. Nelson et al. and Hsia et al. noted the important role of the protocol in referring emergency patients [46] and assisting in the proper sterilization of equipment [45], respectively. The possibility of accessing protocols and guidelines is essential for updating the knowledge of general practitioners in the face of emergencies and their prompt referral [21]. Protocols and guidelines can play a vital role in updating providers’ knowledge and timely referral to higher levels [47]. Their inaccessibility and unavailability will lead to problems in managing LTEs [41, 48].

The third sub-domain in the input dimension was “medical supplies and equipment”, with 57 items as the most important factor in providing better emergency care to patients. Depending on the patient’s emergency needs, the skills of the clinical staff, and the distance from the nearest emergency department, the medical equipment required to manage LTEs should be predicted. The ability to provide basic emergency care in this situation depends significantly on having access to and ensuring their proper functioning [21]. As its deficiency impairs the effective and timely care of patients and may cause irreparable damage [40]. Medical supplies and equipment should be in accordance with the real needs of the centers and in proportion to the qualified staff and should be provided with excellent quality and a sufficient amount of limited resources, on time and at a good and reasonable price [49].

The support equipment is used to diagnose diseases and disinfect other equipment for reuse. Diagnostic equipment such as radiology, electrocardiogram, and others. An electrocardiogram is used to help diagnose myocardial infarction, dysrhythmia, and other heart conditions. Therefore, ensuring the performance of the electrocardiogram in all centers for the evaluation and management of heart patients is essential. Necessary equipment for sterilization such as autoclave, hot-air oven, dry heat or four, and so on. As a result, this equipment can significantly improve the ability to provide primary emergency care in primary healthcare centers and clinics.

The human resource was the fourth sub-domain in the input dimension, which was one of the most basic components of health care systems. In this sub-domain, it is essential to consider the number of staff, their proportions, and the list of emergency teams (physicians and nurses) trained in first aid and CPR. The members of the emergency care team must have a strong theoretical and practical basis in intensive care because in many cases the patient or the injured person’s reaction can be related to the way the staff interacts with the patient and his family [3]. In order to provide primary emergency care, efficient and experienced manpower should be employed, and saving the patient’s life in the shortest time should be a priority of measures and care [50].

Essential emergency medicines were the fifth sub-domain in the input dimension that affect the provision of effective and comprehensive emergency care. This includes emergency medications used for anesthesia, fever, inflammation, infections, poisoning, and more. The World Health Organization has provided a list of essential medicines for all health centers in dealing with emergencies in the Trauma Care Guideline. The presence of emergency medications and anticipation of minimum medication items in accordance with standards and guidelines will save the patient’s life [51, 52] Also, the list of emergency medications for all health centers is defined in the Trauma Care Guideline [4].

Processes a set of activities with clear goals that are supported by resources to achieve the desired outputs [53]. Aspirin for myocardial infarction and brian stroke; evaluation of vital signs in patients with heart attack and sublingual nitroglycerin; basic management of airway, respiration, circulation, and shock; and three-way dressing of vacuum wounds on the chest have saved millions of lives in developed countries [54–57]. Therefore, providing clinical interventions for timely resuscitation management is considered the main service process and as the sixth sub-domain in the input dimension. Managing potentially LTEs requires special knowledge and skills that must be regularly updated and practiced [50, 51]. This is not really easy for NHHC providers who only deal with these conditions occasionally, they may not know how to operate properly, even if basic equipment is available [58, 59]. Because LTEs may occur in the geographical area of ​​operation of these centers, it is expected that these centers will be prepared at least for the initial management of LTEs and the provision of pre-hospital primary care. As a result, the prepardness of these centers for rapid response by trained providers is essential [60]. At the individual level, assessment of skills related to providing clinical interventions for resuscitation management was reported by the center physicians themselves.

The capability to store medicines was the seventh sub-domain in the process dimension that is of great importance for the ability of NHHCs to provide safe and timely emergency care and it is vital that the conditions of drug storage and drug monitoring assess the preparedness of centers. Hsia et al. showed that the storage conditions of drugs should be evaluated by considering that the drugs are located in dry places away from moisture, sunlight, rodents, and pests. They also showed that the number of expired drugs, the stock of drugs based on the expiration date, and the availability of an up-to-date list of available drugs are components that should be evaluated in drug monitoring, and the results of this evaluation indicate the preparedness or unpreparedness of centers [42].

Capability to store medicines was the seventh sub-domain in the process dimension. Ensuring the availability and proper operation of equipment depends on the support, procurement, and planning process, which is also essential for the proper functioning of a healthcare system. Hsia et al. Considered two criteria for evaluating equipment maintenance. One is the existence of a system for repairing buildings or infrastructure and the procedure for their maintenance, and the second is the sterilization or disinfection of equipment for reuse. The second criterion was assessed by the availability of the necessary resources for the sterilization or disinfection of equipment as well as the basic knowledge of employees on the procedure of sterilization or disinfection of equipment [34].

Maintenance of equipment was the eighth sub-domain in the process dimension. Ensuring the availability and proper operation of equipment depends on the support, procurement, and planning process, which is also essential for the proper functioning of a healthcare system. Hsia et al. considered two criteria for evaluating equipment maintenance. One is the existence of a system for repairing buildings or infrastructure and the procedure for their maintenance, and the second is the sterilization or disinfection of equipment for reuse. The second criterion was assessed by the availability of the necessary resources for the disinfection of equipment as well as the basic knowledge of employees on the procedure of sterilization of equipment [42].

Management processes were the ninth sub-domain in the process dimension, which is the responsibility of senior managers of the centers. This process helps prepare NHHCs to better manage LTEs and provide basic emergency care. These processes include continuing education and training, controlling the quality of the referral system, auditing mortality, and risk management. Continuing education is important not only for patient safety but also for motivating providers, especially in rural areas. On the other hand, it is also necessary to develop programs to improve access to emergency care. In Germany, NHHC emergencies occur on average once a month and in rural areas more often than in urban areas. In general, phscicians of NHHCs from rural and urban areas do not face an emergency every day, however, they feel confident in managing various emergency situations. Among physicians, self-confidence may increase with emergency situations that are associated with insecurity, and this also relates to training in pediatric and psychiatric emergencies and regular CPR training, whether or not they have the necessary knowledge and skills. Continuing education programs tailored to the needs of providers in non-hospital centers should be designed to improve their emergency skills. The design of these programs will encourage providers to regularly attend training sessions and increase their overall confidence in dealing with emergency situations [61]. Mortality auditing and risk management allow managers to evaluate the care they currently provide and track their progress in providing good and safe care. Quality control of the referral system is an important issue that should be invested in to strengthen the ability of centers to provide emergency care.

Globally, emergency care delivered in NHHCs has been neglected, with particular emphasis on low- and middle-income countries [62]. Strengthening the emergency care system at the level of non-hospital centers is an urgent issue in many healthcare systems in the world, and there is a need to develop strategic plans in order to reduce mortality and disability [63, 64]. There are multiple complexities in the emergency care chain at the NHHCs level, including poorly resourced emergency areas, inadequate emergency transport, critical personnel shortages, unclear referral pathways, and barriers along the way to escalate emergency patients to appropriate facilities [5]. When there is no support to provide emergency care, healthcare practitioners remain vulnerable [5]. To reduce the inefficiencies, there is a need to review the policies and guidelines to direct emergency care in NHHCs. Supporting emergency care at this level requires the input of experts in the field to provide direction and develop a coordinated emergency care system that addresses the complexities of the chain at each level.

## Conclusion

Iran is a large country with many NHHCs that are responsible for promoting health in the community. Part of this task includes prevention and the other part includes the basic management of emergencies. It is necessary to evaluate the services in each of these centers and to ensure the uniformity of the quality and standards of the services provided [29]. In this study, the developed toolbar help to assess the preparedness of NHHCs in response to LTEs, as well as eliminate existing deficiencies and encourage policymakers, managers, and clients to use these potential capacities [29].

This toolbar was prepared and designed according to the needs of the vice chancellery for health of East Azerbaijan Province and due to the lack of toolbar to assess the preparedness of NHHCs. Using this tool, measurement of all centers can be done in the same way and it is possible to compare centers in this regard. The results of this study led to the development of a valid and reliable toolbar that the vice chancellery for the health of the province can use in monitoring and evaluating the centers. The results of the evaluations will help the center managers in analyzing the situation and comparing their performance with other similar centers.

The current study has some limitations that are mentioned. Future research should consider evaluating toolbar across diverse cultural content. Due to the fact that this study was conducted in East Azerbaijan Province, Iran. The results may not be fully generalizable to other provinces of Iran. Therefore, it is necessary for the vice chancellery for the health of other provinces to adapt the toolbar culturally before using it and use it according to their own needs. Although the NHHC evaluation toolbar does not require exploratory and confirmatory factor analysis, a major limitation in our study was that we were unable to assess structure validity, exploratory factor analysis, and confirmatory factor analysis due to the small sample size and limited financial resources. It is suggested that a study with a larger sample size be designed and conducted to test the validity of the structure and to analyze the exploratory and confirmatory factors of the toolbar. One of the strengths of this toolbar was that various methods were used to extract the items included in the toolbar, including a literature systematic review, SSIs with providers in NHHCs and FGDs with specialists, holding a panel of experts, and so on.

## Supplementary Information

**Additional file 1: **Instrument and manual instrument guide.

## Data Availability

The datasets used and/or analyzed during the current study are available from the corresponding author upon reasonable request.

## Abbreviations

NHHC: Non-hospital Health Center
LTE: Life-threatening Emergency
EMS: Emergency Medical Services
MMAT: Mixed Methods Appraisal Tool
BKNS: Barakat Knowledge Network Systems
SID: Scientific Information Database
SSI: Semi-Structured Interview
FGD: Focus Group Discussion
CVR: Content Validity Ratio
PHC: Primary Health Care
CPR: Cardio Pulmonary Resuscitation
