# Supplementary material for: Non-hospital healthcare center’s preparedness assessment toolbar for providing basic emergency care: a sequential exploratory mixed-method study
Source: BMC Health Serv Res. 2023 Jan 23;23:70. doi: 10.1186/s12913-023-09053-y (PMC9872316; doi:10.1186/s12913-023-09053-y)
Supplement: Supplementary file 1 — Additional file 1: Instrument and manual instrument guide. [file 12913_2023_9053_MOESM1_ESM.docx]

**Instrument and manual instrument guide**


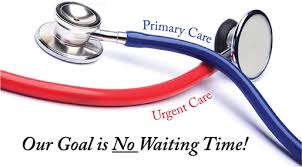


Instrument for Assessing Non-Hospital Health Centers’ Preparedness to Provide Initial Emergency Care

**Instrument for Assessing Non-Hospital Health Centers’ Preparedness to Provide Initial Emergency Care**

| **Center section** | | | |
| --- | --- | --- | --- |
| **Demographic information** | | | |
| **Name of health care center (including city and province):** | | | |
| **Date of data collection:** | | | |
| **Type of health care centers:**  **Urban health centers □**  **Rural health centers □**  **24-hour clinic □** | | | |
|  | **In the past year** | **In the last month** | **In the past week** |
| **Total number of patients referred to this center** |  |  |  |
| **Number of patients referred with life-threatening emergencies** |  |  |  |
| **Number of patients referred due to road traffic injuries** |  |  |  |
| **Number of trauma patients** |  |  |  |
| **The number of patients referred from this center to higher levels** |  |  |  |
| **Number of adjusted death according to severity of injury** |  |  |  |
| **Number of successful CPRs at the center** |  |  |  |

| **Environmental Infrastructures of Centers** | **0** | **1** | **2** | **3** | | |
| --- | --- | --- | --- | --- | --- | --- |
| 1. **Existence of a protective ramp with a slope of 7% angle so that a stretcher or wheelchair passes through it in front of the center entrance door** |  |  |  |  | | |
| 1. **Existence of a special place on the ground floor and near the entrance door of the center to provide emergency care** |  |  |  |  | | |
| 1. **Existence of a waiting room that is managed continuously and regularly under the supervision of a member of clinical staff** |  |  |  |  | | |
| 1. **Existence of emergency power system** |  |  |  |  | | |
| **Protocols, Guidelines and Policies** |  |  |  |  | | |
| 1. **Existence of a written guideline for triage of emergency patients** |  |  |  |  | | |
| 1. **Existence of a written guideline for referral of emergency patients** |  |  |  |  | | |
| 1. **Existence of a written guideline for transfer and dispatch of emergency patients** |  |  |  |  | | |
| 1. **Existence of a written guideline for dealing with common emergencies** |  |  |  |  | | |
| 1. **Existence of a written protocol for the management of multi-trauma patients** |  |  |  |  | | |
| 1. **Existence of a risk management policy** |  |  |  |  | | |
| 1. **Existence of written policy and procedure on how to use and maintain medical equipment** |  |  |  |  | | |
|  |  |  |  |  | | |
| **Medical Supplies and Equipment** | **0** | **1** | **2** | **3** | | |
| 1. **Portable suction with acceptable suction (in addition to central suction), suction interface and Nelaton or Yanquire suction** |  |  |  |  | | |
| 1. **Neonatal and pediatric laryngoscope with blades in different curved and smooth sizes (with 2 healthy spare batteries and one laryngoscope spare lamp)** |  |  |  |  | | |
| 1. **Adult laryngoscopes with different curved and smooth sizes (with 2 healthy spare batteries and one laryngoscope spare lamp)** |  |  |  |  | | |
| 1. **Ambobag (BVM)** |  |  |  |  | | |
| 1. **Stethoscope** |  |  |  |  | | |
| 1. **Pediatric stethoscope** |  |  |  |  | | |
| 1. **Oxygen capsule with capsule carrier wheel and protective chain and oxygen interface tube** |  |  |  |  | | |
| 1. **Blood pressure cuff** |  |  |  |  | | |
| 1. **Peak flowmeter** |  |  |  |  | | |
| 1. **X-ray** |  |  |  |  | | |
| 1. **EKG** |  |  |  |  | | |
| 1. **Ultrasound device** |  |  |  |  | | |
| 1. **Otoscope for ear examination** |  |  |  |  | | |
| 1. **Ophthalmoscope for eye examination** |  |  |  |  | | |
| 1. **Autoclave** |  |  |  |  | | |
| 1. **Dry heat or four** |  |  |  |  | | |
| 1. **Forceps** |  |  |  |  | | |
| 1. **Magill forceps** |  |  |  |  | | |
| 1. **Tourniquet** |  |  |  |  | | |
| 1. **Essential dressing set** |  |  |  |  | | |
| 1. **Dressing table** |  |  |  |  | | |
| 1. **Chest Tube set** |  |  |  |  | | |
| 1. **Thermometer** |  |  |  |  | | |
| 1. **Portable iv stand** |  |  |  |  | | |
| 1. **Safety Box** |  |  |  |  | | |
| 1. **Disposable mask** |  |  |  |  | | |
| 1. **Gloves** |  |  |  |  | | |
| 1. **Goggles** |  |  |  |  | | |
| 1. **Scrubs** |  |  |  |  | | |
| 1. **Infectious and non-infectious waste bins** |  |  |  |  | | |
| 1. **Suture set** |  |  |  |  | | |
| 1. **Tracheostomy set** |  |  |  |  | | |
| 1. **Scissors** |  |  |  |  | | |
| 1. **Glasgow or GCS Coma Scale Sheet (Head Shock)** |  |  |  |  | | |
| 1. **Reflex hammer for examination** |  |  |  |  | | |
| 1. **Sling** |  |  |  |  | | |
| 1. **Splint** |  |  |  |  | | |
| 1. **Long backboard** |  |  |  |  | | |
| 1. **Sandbag (spinal cord injury)** |  |  |  |  | | |
| 1. **Broselow pediatric emergency tape** |  |  |  |  | | |
| 1. **Emergency trolley; Available to clinical staff so that resuscitators can use its contents in less than a minute** |  |  |  |  | | |
|  | | | | | |  |
|  | **0** | **1** | **2** | **3** | **4** | |
| 1. **Silk threads 2-0 to 6-0 cut into lengths of 0.5 mm to 3 cm(at least 3 pieces)** |  |  |  |  |  | |
| 1. **Nasal Cannula (at least 3)** |  |  |  |  |  | |
| 1. **Urine Bag (at least 3)** |  |  |  |  |  | |
| 1. **Oxygen interface (at least 3 pcs)** |  |  |  |  |  | |
| 1. **Suction interface tee (at least 3)** |  |  |  |  |  | |
| 1. **Angiocath- Three Way Stop Cock(at least 3)** |  |  |  |  |  | |
| 1. **Foley Catheter** |  |  |  |  |  | |
| 1. **NG-tube** |  |  |  |  |  | |
| 1. **Cuffed endotracheal tube 4, 5, 6, 7, 7/5 and 8/5** |  |  |  |  |  | |
| 1. **Uncuffed Endotracheal Tubes** |  |  |  |  |  | |
| 1. **Oral Airway** |  |  |  |  |  | |
| 1. **Nasal Airway** |  |  |  |  |  | |
| 1. **Angiocath** |  |  |  |  |  | |
| 1. **Types of Scalp Vein** |  |  |  |  |  | |
| 1. **Types of syringes 2, 5 and 10, 20, 50 cc** |  |  |  |  |  | |
| 1. **Disposable face oxygen mask (Face Mask) with interface tube in children and adults sizes of at least one** |  |  |  |  |  | |
|  | **0** | **1** | **2** | **3** | | |
| **Human Resources** |  |  |  |  | | |
| 1. **Resident physician** |  |  |  |  | | |
| 1. **Nursing staff** |  |  |  |  | | |
| 1. **Have a list of clinical staff (physicians and nurses) trained in first aid and cardiopulmonary resuscitation** |  |  |  |  | | |
|  | **0** | **1** | **2** | **3** | | |
| **Emergency Medicines** |  |  |  |  | | |
| 1. **Lidocaine** |  |  |  |  | | |
| 1. **Diazepam** |  |  |  |  | | |
| 1. **Atropine Sulfate** |  |  |  |  | | |
| 1. **Acetaminophen Codeine** |  |  |  |  | | |
| 1. **ASA** |  |  |  |  | | |
| 1. **Ibuprofen** |  |  |  |  | | |
| 1. **Dexamethasone phosphate** |  |  |  |  | | |
| 1. **Hydrocortisone** |  |  |  |  | | |
| 1. **Epinephrine** |  |  |  |  | | |
| 1. **Naloxan Hydrochloride** |  |  |  |  | | |
| 1. **Phenobarbital Sodium** |  |  |  |  | | |
| 1. **Phenytoin Sodium** |  |  |  |  | | |
| 1. **MgSO4 50%** |  |  |  |  | | |
| 1. **Ampicillin** |  |  |  |  | | |
| 1. **Co-amoxiclav** |  |  |  |  | | |
| 1. **Penicillin G (Na,K)** |  |  |  |  | | |
| 1. **Chloramphenicol** |  |  |  |  | | |
| 1. **Ciprofloxacin** |  |  |  |  | | |
| 1. **Cloxacillin Sodium** |  |  |  |  | | |
| 1. **Gentamicin** |  |  |  |  | | |
| 1. **Metronidazole** |  |  |  |  | | |
| 1. **Sulfamethoxazole** |  |  |  |  | | |
| 1. **Trimethoprim** |  |  |  |  | | |
| 1. **Furosemide** |  |  |  |  | | |
| 1. **Mannitol 20%** |  |  |  |  | | |
| 1. **Sodium Chloride 5%** |  |  |  |  | | |
| 1. **Sodium Chloride 0.9%** |  |  |  |  | | |
| 1. **Ringer lactate** |  |  |  |  | | |
| 1. **Potassium Chloride** |  |  |  |  | | |
| **Individual section** | | | | | | |
|  | **0** | **1** | **2** | **3** | | |
| **Clinical Interventions** |  |  |  |  | | |
| 1. **Registering the severity of injury (At least as (GAP) (GCS, Age & Pluse p))** |  |  |  |  | | |
| 1. **Head tilt/Chin lift** |  |  |  |  | | |
| 1. **Oropharyngeal Airway** |  |  |  |  | | |
| 1. **Nasopharyngeal Airway** |  |  |  |  | | |
| 1. **Use of suction** |  |  |  |  | | |
| 1. **Ventilation using bag–valve–mask** |  |  |  |  | | |
| 1. **Needle cricothyrotomy** |  |  |  |  | | |
| 1. **Three-way dressing** |  |  |  |  | | |
| 1. **IV therapy** |  |  |  |  | | |
| 1. **Inserting IV cannula** |  |  |  |  | | |
| 1. **Inserting urinary catheters** |  |  |  |  | | |
|  | **0** | **1** | **2** | **3** | | |
| **Medicine Storage Capability** |  |  |  |  | | |
| 1. **Maintaining the storage conditions of emergency medicines** |  |  |  |  | | |
| 1. **Putting emergency medicines in a separate medicine cabinet** |  |  |  |  | | |
| 1. **Arrangement of drugs in the emergency trolley** |  |  |  |  | | |
| 1. **Outdated drugs** |  |  |  |  | | |
| 1. **An up-to-date list of essential emergency medications** |  |  |  |  | | |
| 1. **Existence of routine schedule for requesting pharmaceutical items from relevant organizations or supply by the centers themselves** |  |  |  |  | | |
|  | **0** | **1** | **2** | **3** | | |
| **Maintenance of equipment** |  |  |  |  | | |
| 1. **Repair and maintenance of equipment by reputable companies** |  |  |  |  | | |
| 1. **Calibration test of important equipment** |  |  |  |  | | |
| 1. **Existence of a specific program for repair, maintenance and calibration of medical center equipment** |  |  |  |  | | |
|  | **0** | **1** | **2** | **3** | | |
| **Management process** |  |  |  |  | | |
| 1. **Patient referral** |  |  |  |  | | |
| 1. **Transportation and collection of infectious waste** |  |  |  |  | | |
| 1. **Meetings held to investigate deaths related to casualties with life-threatening emergencies** |  |  |  |  | | |
| 1. **Risk assessment by one of two methods (RCA or FMEA)** |  |  |  |  | | |
| 1. **Continuing education courses** |  |  |  |  | | |
| 1. **Documents for holding continuous training courses for human resources personnel** |  |  |  |  | | |

**Manual Instrument Guide** **for Assessing Non-Hospital Health Centers’ Preparedness to Provide Initial Emergency Care**

**A guide to using tools in evaluations and how to score items.**

**The present guide was designed and prepared to help evaluators to better evaluate.**

**Evaluations are generally at both the center and individual levels before the evaluation. At the individual level, the skills of the clinical staff of the centers (physicians) are evaluated in 11 service delivery processes, and at the center level, the remaining questions are evaluated.**

**Life-threatening emergency (LTE) life-threatening emergency is defined as an imminent life-threatening situation that requires the establishment of special resources and measures to address this situation.**

1. Epilepsy
2. Acute stroke
3. Abrasion of the cornea
4. Foreign body in the eye
5. F. body in ENT
6. Epistaxis
7. Fracture
8. Dislocation
9. Cut wound
10. Anaphylactic reaction
11. Cardiac arrest
12. Hypertension emergencies
13. Shock
14. Acute dyspnea
15. Chest pain
16. Palpitation
17. Acute bronchial asthma
18. Acute hemolysis
19. SCD crisis
20. GIT bleeding
21. Acute abdomen
22. Hypoglycemia
23. Hyperglycemic emergencies
24. Renal colic
25. Acute urine retention
26. PV bleeding
27. Abdominal pain in pregnancy
28. Burns
29. Chemical accident
30. Animal bites and stings

**How to score**

**From items 1 to 11**

**Score 0:** Not available.

**Score 1:** Available.

**From items 12 to 27**

**Score 0:** Not available.

**Score 1:** Available, but not ready for immediate use.

**Score 2:** Available and ready for quick use.

**From items 28 to 51**

**Score 0:** Not available.

**Score 1:** Available.

**Item 52: Emergency trolley; Available to clinical staff so that resuscitators can use its contents in less than a minute**

**The assessor asks randomly the care staff of the center to place the trolley next to the bed and make sure that there is no obstruction in its passage, the trolley moves easily and it can be placed next to the bed for less than one minute.**

**Score 0:** Even if one of the conditions is not met.

**Score 1:** At least half of the mentioned conditions are met**.**

**Score 2:** The maximum conditions mentioned are observed.

**From items 53 to 68**

**Score 0:** None

**Score 1:** No variety, insufficient number.

**Score 2:** No variety, enough number.

**Score 3:** Variety, insufficient number.

**Score 4:** Variety, enough number.

**Item 69: Resident Physician**

**Score 0:** The number of physician according to the standard is not enough**.**

**Score 1:** The number of physician according to the standard is sufficient.

**Item 70: Nurse**

**Score 0:** The number of nurse according to the standard is not enough**.**

**Score 1:** The number of nurse according to the standard is sufficient.

**Item 71: Have a list of clinical staff (physicians and nurses) trained in first aid and cardiopulmonary resuscitation.**

**Score 0:** Not available.

**Score 1:** Available.

**Items 72 to 100: The assessor, according to who guideline standards, thus rates the emergency medications available at the center.**

**Score 0:** Not available.

**Score 1:** Insufficient one number.

**Score 2:** Almost enough for at least half of the judges.

**Score 3:** Enough.

**Attention!!! Points should be given to the preferred referees in this way.**

**Score 0:** Not available.

**Score 1:** Available.

**.**

| **Drug form** | **Medicine name** | **Description** |
| --- | --- | --- |
| **Amp. 5 ml** | **Lidocaine Hydrochloride 2%** |  |
| **Amp. 5 mg/ml, 2 ml** | **Diazepam** |  |
| **Amp. 0.5 mg/ml** | **Atropine Sulfate** |  |
| **Tab. 300/10** | **Acetaminophen Codeine** | **Preferred drugs** |
| **Tab. 100 mg** | **ASA** |  |
| **Amp. 4 mg/ml, 2 ml** | **Dexamethasone phosphate** |  |
| **Amp. 50 mg/2 ml** | **Hydrocortisone** |  |
| **Amp. 1 mg/ml, 2 ml** | **Epinephrine** |  |
| **Amp. 0.4 mg/ml** | **Naloxan hydrochloride** |  |
| **Amp.200mg/ml,1ml** | **Phenobarbital Sodium** |  |
| **Amp. 50 mg/ml,5ml** | **Phenytoin Sodium** |  |
| **Vial 50 ml** | **MgSO4 50%** |  |
| **Vial 500 mg, 1 gr** | **Ampicillin** | **Preferred drugs** |
| **Tab** | **Co-amoxiclav** |  |
| **Vial 5,000,000 IU** | **Penicillin G (Na,K)** |  |
| **Tab. 250 mg** | **Chloramphenicol** |  |
| **Vial 2 mg/ml, 100 ml** | **Ciprofloxacin** | **Preferred drugs** |
| **Vial 250, 500 mg** | **Cloxacillin Sodium** | **Preferred drugs** |
| **Amp. 40 mg/ml, 2 ml** | **Gentamicin** | **Preferred drugs** |
| **Vial 5 mg/ml, 100 ml** | **Metronidazole** | **Preferred drugs** |
| **Tab. 250 mg** | **Metronidazole** |  |
| **Tab. 400/80mg** | **sulfamethoxazole** | **Preferred drugs** |
| **Tab. 100mg** | **Trimethoprim** | **Preferred drugs** |
| **Amp. 10 mg/ml,2ml** | **Furosemide** |  |
| **Vial 500 ml** | **Mannitol 20%** |  |
| **Vial 50 ml** | **Sodium Chloride 5%** |  |
| **Irrigation 1000 ml** | **Sodium Chloride 0.9%** |  |
| **Solution 500ml, 1000 ml** | **Ringer lactate** |  |
| **Vial 2 meq/ml,50 ml** | **Potassium Chloride** |  |

**Item 101: Registering the severity of Injury (At least as (GAP) (GCS, Age & Pluse p))**

**Score 0:** Not registered.

**Score 1:** Low quality is registered.

**Score 2:** Registered but not for almost everyone.

**Score 3:** High quality and registered for everyone.

**From item 102 to 111: The evaluator gives the clinical staff a score from 0 to 4 based on the conditions that must be observed for each of the clinical processes and interventions.**

**Score 0:** I'm not sure at all

**Score 1:** I'm somewhat confident

**Score 2:** I'm sure

**Score 3:** I'm absolutely sure

**Item 112 "Maintaining the storage conditions of emergency medicines"**

**Requirements:**

Emergency medicines should be stored in a cool, dry place away from moisture, sunlight and pests in accordance with the storage standards of the medicine.

**Score 0:** If emergency medications are not stored according to the mentioned standard**.**

**Score 1:** If it is maintained to some extent according to the mentioned standard**.**

**Score 2:** Maintain completely according to the mentioned standard.

**Item 113: Emergency medicines are on a separate shelf**

**Score 0:** Not available.

**Score 1:** Available.

**Item 114 "Arrangement of drugs in the emergency trolley"**

**Requirements:**

**How to arrange the medicine in the emergency trailer from top to bottom according to the following conditions:**

- **First floor: emergency medicines**
- **Second floor: all kinds of syringes, oral and nasal airway, bandages and sterile gauze, examination gloves**
- **Third floor: angiocath, scalp vein, serum**
- **Fourth floor: laryngoscope, chip tubes, ambobag, sethoscope and sphygmomanometer, Nelaton and Foley catheter.**

**In order to prevent incorrect injection of drugs with similar forms (especially vials of magnesium sulfate, dextrose, etc.), these drugs should be distinguished with appropriate labeling.**

**Score 0:** The mentioned conditions are not observed**.**

**Score 1:** Somewhat observed**.**

**Score 2:** Fully complied with.

**Item 115: Number of expired drugs"**

Ten drugs are randomly evaluated by evaluators

**Score 0:** If there is a case of expired drug, a score of zero is given (if the expired drug has been isolated and minutes have been taken during the last month).

**Score 1:** If there are no outdated drugs.

**Item 116: An up-to-date list of essential emergency medications**

**Score 0:** If not available

**Score 1:** If available

**Item 117: Existence of routine schedule for requesting pharmaceutical items from relevant organizations or supply by the centers themselves**

**Score 0:** If not available

**Score 1:** If available

**Item 118: Repair and maintenance of equipment by reputable companies Score**

**Score 0:** It is not done by reputable companies**.**

**Score 1**: If it has been done by reputable companies but the relevant documents are not available**.**

**Score 2:** If it has been done by reputable companies and their related documents are available**.**

**Item 119: Calibration test of important equipment**

**Score 0:** If the calibration test of important equipment has not been performed**.**

**Score 1:** If the calibration test has been performed but has not been documented.

**Score 2:** If the calibration test has been performed and documented.

**Item 120: Existence of a specific program for repair, maintenance and calibration of medical center equipment**

**Score 0:** If not available

**Score 1:** If available

**Item 121: Patient referral**

A number of injured cases with life-threatening emergencies are randomly reviewed by assessors.

**Score 0:** If appropriate measures such as examination, timely referral, face-to-face or telephone follow-up, submission of feedback, etc. have not been taken.

**Score 1:** In 50% of cases, appropriate measures such as examination, timely referral, face-to-face or telephone follow-up, submission of feedback, etc. have been performed.

**Score 2:** In 100% of cases, appropriate measures such as examination, timely referral, face-to-face or telephone follow-up, feedback, etc. have been performed.

**Item 122: Transportation and collection of infectious waste**

**Score 0:** Infectious waste has not been transported and collected by municipal motor organizations.

**Score 1:** If the infectious waste has been transported and collected by the municipal motor organizations but the relevant documents are not available.

**Score 2:** If the infectious waste has been transported and collected by the municipal motor organizations and the relevant documents are available.

**Item 123: Meetings held to investigate deaths related to casualties with life-threatening emergencies**

**Score 0:** If not held.

**Score 1:** If it is held at least once every 6 months but the relevant documents are not available.

**Score 2:** If it is held at least once every 6 months and the relevant documents are available.

Note: Statements are not a criterion.

**Item 124: Risk assessment by one of two methods (RCA or FMEA)**

**Score 0:** No risk assessment has been performed at all.

**Score 1:** At least one error has occurred and has been evaluated by one of these two methods, but the relevant documentation is not available.

**Score 2:** At least one error has occurred and has been evaluated by one of these two methods and the relevant documentation is available.

**Item 125: Continuing education courses**

**Score 0:** None of the personnel reported receiving continuing education during the past year.

**Score 1:** At least half of the personnel report receiving training courses during the past year.

**Score 2:** More than half of the staff report receiving training courses during the past year.

**Item 126: Documents for holding continuous training courses for human resources personnel**

**Score 0:** If not available

**Score 1:** If available
